# Supplementary material for: Patterns of Intron Gain and Loss in Fungi
Source: PLoS Biol. 2004 Nov 30;2(12):e422. doi: 10.1371/journal.pbio.0020422 (PMC532390; doi:10.1371/journal.pbio.0020422)
Supplement: Table S1 — Also available at http://genes.mit.edu/NielsenEtAl/. (4.3 MB ZIP). [file pbio.0020422.st001.zip › NielsenEtAl/html/762.html]

AN5912.1.NCU08989.1.MG04976.1.FG06920.1


```
 CLUSTAL W (1.82) Multiple Sequence Alignments - Introns Inserted


Sequence 1: NCU08989.1	183 aa
Sequence 2: MG04976.1	190 aa
Sequence 3: FG06920.1	186 aa
Sequence 4: AN5912.1	182 aa
Alignment Length: 193 aa
Number Identitical Residues: 141 aa
Alignment Score (without introns) 5757


MG04976.1 	MGQSVSWLS---GLIWAKKEIRILILGL0DNAGKTTLLYRLK0IGDVVTTIPTIGFNVES
NCU08989.1	MGQSMSWLSSLSNLFWTKKEIRILILGL~DNAGKTTLLYRLK0IGEVVTTIPTIGFNVES
FG06920.1 	MGNSMSWFS---NLLFSKKEIRILILGL0DNAGKTTLLYRLK0VGEVVTTIPTIGFNVES
AN5912.1  	MGGSLSRLW---SLFWSKKEIRILILGL0DNAGKTTLLYRLK0IGEVVTTIPTIGFNVES
          	** *:* :    .*:::*********** ************* :*:**************

MG04976.1 	VTYKNLNFNVW~DLGGQTSIRPYWRCYYANTAAVIFVVDSTDIDRLQTAADELAAMLNED
NCU08989.1	VTYKNLNFNVW~DLGGQTSIRPYWRCYYANTAAVIFVVDSTDMERLQTAADELAAMLNEE
FG06920.1 	VTYKNLNFNVW0DLGGQTSIRPYWRCYYANTAAVIFVVDSTDIERLHTASEELSAMLNEE
AN5912.1  	VTYRNLNFNVW0DLGGQTSIRPYWRCYYANTAAVVFVIDSTDVERLGTAADELAAMLNEE
          	***:******* **********************:**:****::** **::**:*****:

MG04976.1 	ELKDAALLVFANKQDQPGAKGAGDISQALRLGELRDRNWSIMACSAVDGSGVNEGMDWLV
NCU08989.1	ELKDAALLVFANKQDQPGAKGAADISQALRLGELRDRNWSIMACSAVEGTGITEGMDWLV
FG06920.1 	ELKDAALLVFANKQDQPGAKGAGEISEALRLGELRDRNWSIMACSAVDGSGVNEGMDWLV
AN5912.1  	ELREAALLVFANKQDQPGAKGAGEISEALKLGELRDRNWSIVACSAIDGKGLNEGMDWLV
          	**::******************.:**:**:***********:****::*.*:.*******

MG04976.1 	0QY~QDRGQTLPWVGRLY
NCU08989.1	0QT~VKEE----------
FG06920.1 	~VS1PIEP----WLRFSN
AN5912.1  	0QT~LQSENA--------
          	        .:
```
